# The Number of Platelets in Patient’s Blood Influences the Mechanical and Morphological Properties of PRP-Clot and Lysophosphatidic Acid Quantity in PRP

**DOI:** 10.3390/ijms21010139

**Published:** 2019-12-24

**Authors:** Michela Bosetti, Paolo Boffano, Alice Marchetti, Massimiliano Leigheb, Mattia Colli, Matteo Brucoli

**Affiliations:** 1 Dipartimento di Scienze del Farmaco, University of Eastern Piedmont, L.go Donegani 2, 28100 Novara, Italy; alicemarchetti9@gmail.com (A.M.); mattiacolli@shbclinic.com (M.C.); 2 Division of Maxillofacial Surgery, University of Eastern Piedmont, University Hospital “Maggiore della Carità”, 28100 Novara, Italy; paolo.boffano@gmail.com (P.B.); matteo.brucoli@med.uniupo.it (M.B.); 3 Dipartimento di Scienze della Salute, University of Eastern Piedmont, Via Solaroli 17, 28100 Novara, Italy; massimiliano.leigheb@uniupo.it; 4 Division of of Orthopaedics and Traumatology, University Hospital “Maggiore della Carità”, 28100 Novara, Italy

**Keywords:** PRP, osteoblasts, LPA, bone regeneration, regenerative medicine, growth factors

## Abstract

The objectives of this study were to compare platelet-rich plasma (PRP) from patients with different concentrations of platelets and to assess the influence of these PRP preparations on human osteoblast (hOB) activity. In the literature, growth factors released by activated platelets have been considered responsible for the active role of PRP on bone regeneration but no specific role has been attributed to lysophosphatidic acid (LPA) as a possible effector of biological responses. In this study, patients were grouped into either group A (poor in platelets) or group B (rich in platelets). Clots from PRP fraction 2 (F2-clots), obtained with CaCl_2_ activation of PRP from the two groups, were compared macroscopically and microscopically and for their mechanical properties before testing their activity on the proliferation and migration of hOB. LPA was quantified before and after PRP fractioning and activation. The fibrin network of F2-clots from patients with a lower platelet concentration had an organized structure with large and distinct fibers while F2-clots from patients in group B revealed a similar structure to those in group A but with a slight increase in density. ELISA results showed a significantly higher plasma level of LPA in patients with a higher platelet concentration (group B) in comparison to those in group A (*p* < 0.05). This different concentration was evidenced in PRP but not in the clots. Depending on the number of platelets in patient’s blood, a PRP-clot with higher or lower mechanical properties can be obtained. The higher level of LPA in PRP from patients richer in platelets should be considered as responsible for the higher hOB activity in bone regeneration.

## 1. Introduction

In the field of regenerative medicine and tissue engineering, there is great interest in the search for new compounds to be used as fillers and as promoters of tissue regeneration. The use of platelet concentrates in combination with natural and synthetic biomaterials has been widely assessed and examined, with the aim of obtaining a faster and better bone healing [1]. Platelet-rich plasma (PRP) is a widely used biomaterial for bone tissue regeneration, both as a three-dimensional scaffold with filling function and as a pharmacologically active compound capable of stimulating cell migration and cell proliferation at the site of the lesion [2,3,4]. Other strategies in bone remodeling/repairing exist. For example, dental pulp tissue represents a source of mesenchymal stem cells that have a strong differentiation potential towards the osteogenic lineage and decellularized bone extracellular matrix might be considered as suitable scaffolds to support osteogenic differentiation of dental pulp stem cells [5]. Furthermore, novel strategies to replace bone tissue can be considered too. For example, Kerativitayanan et al. reported synthesis and fabrication of porous and elastomeric nanocomposite scaffolds from biodegradable poly (glycerol sebacate) (PGS) and osteoinductive nanosilicates [6].

Historically, the active role of PRP in biological responses promoting tissue regeneration has been attributed to growth factors released by activated platelets [7,8] whilst the role of lysophosphatidic acid (LPA) has been neglected. Lysophosphatidic acid is a biologically active phospholipid with pleiotropic activity (as a protein growth factor) that is made up of a doubly esterified glycerol molecule. Within the bloodstream, LPA molecules are mainly linked to serum albumin and they exhibit variable physiological plasma levels (0.077 ± 0.026 μM in male subjects; 0.103 ± 0.032 μM in females) [9]. Furthermore, LPA is produced by platelets following a stimulation by prothrombotic agents (e.g., thrombin) [10]; it is also produced by osteoblasts and is potentially involved in bone remodeling mechanisms by regulating osteoblast activity in cooperation with vitamin D and by paracrine regulation of osteoclasts [11,12]. It has recently emerged as a highly significant regulator of bone cell biology by inducing chemotaxis, growth, maturation, and survival of osteoblasts [13,14]; by promoting dendrite outgrowth in osteocytes; and by stimulating osteoblastic differentiation of human mesenchymal stem cells (hMSC) [15,16]. In our recent article, we demonstrated that LPA confers osteoconductive properties to scaffold materials and that it fastens bone fragments through actin cytoskeleton reorganization and myosin light chain phosphorylation of human primary osteoblasts [17].

Therefore, although the active role of PRP has been attributed to the growth factors released by activated platelets, the role of LPA as a possible biologically active compound for bone regeneration should also be considered.

The aim of our study was to quantify the concentration of platelets in PRP samples and their role in the determination of PRP morphological quality. A macroscopic, microscopic, and mechanical analysis of clots formed by platelet activation was performed to highlight the morphology, consistency, and structural diversity of the clot matrices; the adhesion capacity of human primary osteoblast-like cells was determined together with their migration activity. Finally, we focused on the remodeling of extracellular matrix and on the induction of cell proliferation. No specific role has been attributed to LPA in PRPs as a possible effector of biological responses in tissue regeneration so we have quantified LPA in the prepared plasma fractions and plasma clots.

After reviewing the literature on PRP preparation, our work has been focused on the production of PRP fractions by centrifugation using the Endoret™ technique to obtain coagulated fibrin gels [18]. Human blood samples used for PRP production were classified into two groups according to their platelet concentration (poor or rich), a variable that could determine a different quality PRP. Macroscopic analysis highlighted the morphology and the diversity of the clot consistency whereas a microscopic investigation showed the structural diversity of PRP matrices obtained from the two groups, which were then examined for cellular interaction, adhesion capacity, and cell migration.

## 2. Results

**Figure 1:**
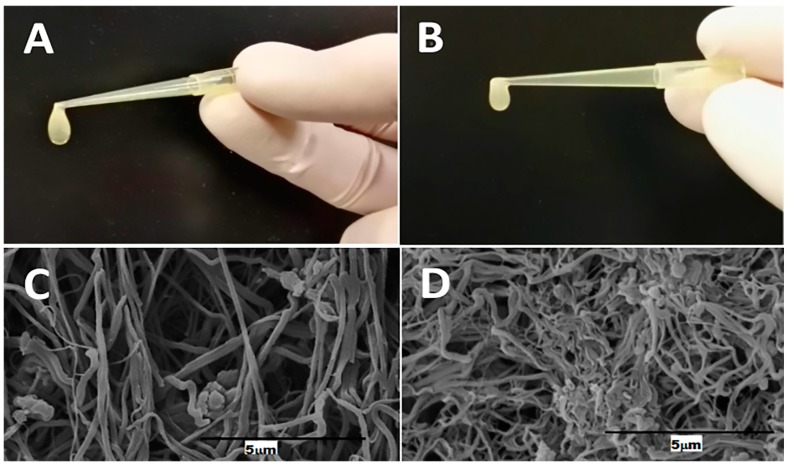
Representative images of F2-clots obtained by activating 150 μL of platelet-rich plasma (PRP)-F2 with CaCl_2_ for 1 h at 37 °C using PRP from patients of group A, with lower platelet concentration ((**A**) macroscopic photograph; (**C**) SEM microscopic photograph) and from patients of group B, with higher platelet concentration ((**B**) macroscopic photograph; (**D**) SEM microscopic photograph).

Few differences were found in the consistency and macroscopic structure of clots obtained by PRP-F2 coagulation with calcium chloride using PRP from patients of group A and patients of group B (Figure 1A,B). In both gels, there was a similar tendency to contract and compact with prolonged handling although there appeared to be higher viscoelastic properties seen in F2-clots from group B.

Electron microscopy of the fibrin scaffold (Figure 1C,D) showed that the fibrin network of F2-clots from patients in group A had an organized structure with large and distinct fibers while F2-clots from patients in group B revealed a similar structure but with an increase in density of the network. We might hypothesize that the increased density of the fibrin observed in F2-clots from group B could be associated with the higher quantity of platelets in PRPs and that the release of fibrinogen contained in their granules following activation could be speculated.

**Figure 2:**
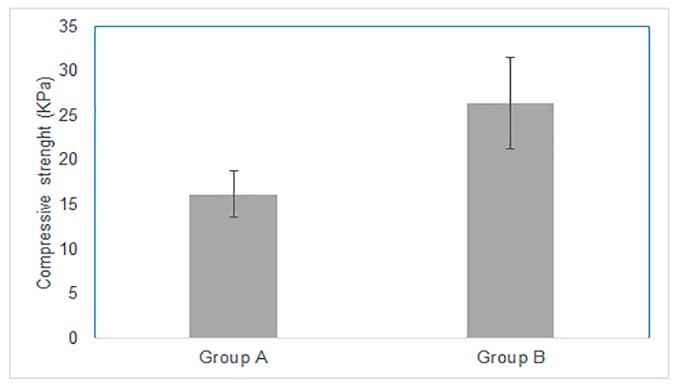
Histogram reporting the compressive strength of the F2-clots from the two groups of patients (mean ± SD; *n* = 8; *p* > 0.05).

The mechanical properties of the F2-clots evaluated by compression are shown in Figure 2. There was no significant difference between the groups (*p* > 0.05) although clots from PRP of the patients in group B (higher in platelets) had higher compressive strength (26 ± 5 KPa) than those of group A (16 ± 2 KPa).

**Figure 3:**
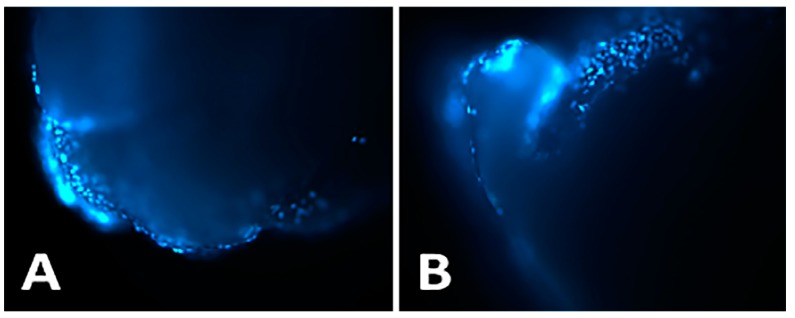
Fluorescence dye images showing DAPI-positive human osteoblasts (hOBs) adhering to the surface of F2-clots from group A (**A**) and group B (**B**). Images acquired with a 4× objective.

Assays of cell adhesion to materials used as fillers are indicative of whether cells find an environment conducive to their local development and, therefore, are useful tests to understand how cells may promote the integration of the material into the location of the damage. Fluorescence microscopy showed that cells on the outside surface of F2-clots were high in density and well distributed, with no differences between the two groups of patients (Figure 3A,B).

**Figure 4:**
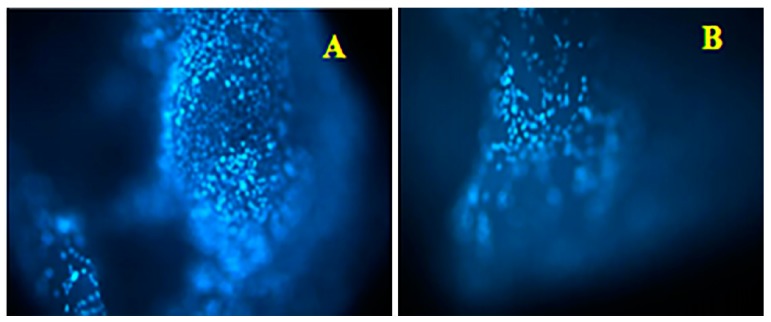
Fluorescence dye images representative of hOBs that populated the inner part of the F2-clots from group A (**A**) and group B (**B**). Images acquired with a 4× objective.

Cell migration assays in materials used as fillers are an index of an environment conducive to tissue regeneration with matrix remodeling at the site of the damage. Platelet-rich plasma is an autologous biomaterial and is expected to show good cell adhesion; moreover, being reasonably porous and pharmacologically active, thanks to the presence of growth factors, it is also expected to favor internal colonization when hosted in vivo and to develop a host-like tissue. Sagittal cuts of the F2-clots were made to see the 3D scaffold’s internal structure, and images were captured and evaluated to study cell migration. Representative images of DAPI-stained cells are shown (Figure 4A,B).

**Figure 5:**
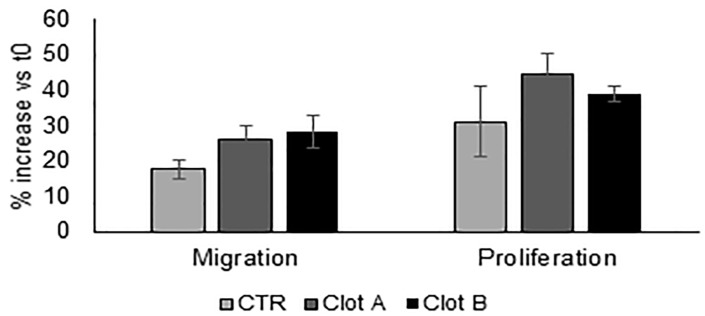
Histogram reporting proliferation and migration activity on the monolayer of hOB. *n* = 8, (mean ± SD; *n* = 10; *p* > 0.05).

Comparing images, the two groups of patients showed similar results with good cells penetration into the scaffolds. Although the clots had different geometric shapes and different thickness, it seems that the fibrin mesh underneath provided a structure that determines the direction of cell accession. After evaluating almost 10 sections from each clot, we have seen that there were more cells populating the inner part of the scaffold in group A (poorer in platelets). The F2-clots produced an increase in human osteoblast proliferation compared to the control which was not statistically significant (*p* > 0.05); they also promoted hOB cell migration. As shown in the histogram in Figure 5, both clot groups showed that filling of the denuded areas on the dish with cells was greater than in the untreated controls.

When compared to the control, monolayers of osteoblasts treated with PRP-clots exhibited a 10% higher increase in the average distance that the cells moved over the course of the experiment. No differences were seen between the two groups, indicating similar activity on hOB migration of clots obtained from the two groups of subjects.

The high heterogeneity of platelets number in our 37 subjects is as expected for physiological data with normal values ranging between 150,000 and 400,000 units/μL.

The 37 patients were divided in two groups based on their platelet number: group A patients with 114,500 ± 35,500 platelets and group B with 232,600 ± 55,600 platelets. Then, the total amount of platelets in the two plasma fractions (F1 and F2 obtained by centrifugation) was determined, demonstrating the tendency of platelets to be approximately twice as concentrated in the F2 fraction (Table 1).

Platelet number was considered important in the study as it was hypothesized that the number of platelets could affect the quality of the clot, the basal LPA plasma level, and the amount of LPA released during activation and degranulation.

The ELISA results showed a higher plasma LPA level in the group of patients with higher platelet concentrations (group B) with statistical significance when compared to group A (*p* < 0.05). Centrifugation to obtain PRP did not alter the LPA concentration, and no differences were seen between F1 and F2 within a group, as shown in Table 2.

The LPA value in the lysate of PRP was expected to increase after coagulation and therefore platelet activation, with a higher increase in clots of group B being higher in platelets, but this was not found. Levels of LPA in lysates of F2-clots were lower than the levels in F2 before CaCl_2_ activation and were similar in the two different study groups despite the large difference in platelet number between the two study groups. Therefore, the activation of platelet-rich plasma with calcium chloride did not increase LPA levels. Following clotting, a slight decrease in the residual (supernatant) plasma LPA levels in comparison with the initial plasma values was noticed. However, such decrease seemed to be proportional to the initial values for each group and was dependent on the number of platelets in the group. Therefore, LPA levels were not increased by CaCl_2_ activation.

## 3. Discussion

An initial review of the literature highlighted the great variety of platelet-derived products and the heterogeneity in their nomenclature and processing methods, with confusion over their therapeutic properties and suggested applications [18,19,20,21]. Most protocols of PRP preparation are very similar to each other: the withdrawal of autologous blood in the presence of an anticoagulant; its centrifugation with plasma separation; leukocyte inclusion or exclusion; and the activation of platelet with different stimuli. However, current reporting of PRP preparation and composition does not enable a comparison of the PRP products delivered to patients.

Despite the variety of commercial products offered to obtain PRPs, two major groups can be identified: (1) PRPs (platelets-rich plasma), F1, and F2 in our study as suggested by Anitua et al. [22] and (2) PRFs (platelet-rich plasma rich in fibrin) and F2-clot in our study. Moreover, these two groups have sometimes been used enriched in leukocytes (L-PRP and L-PRF), but they were not considered in our study. Together with these differences in PRP preparation protocols and PRP composition that do not allow comparison between bony defects clinical trials, to our knowledge, nobody has considered platelet number and platelet activity as individual heterogenous variables that could affect PRP quality and activity.

Most in vitro studies conducted on PRP have focused on the effects of activated PRP rather than on platelet gel and its individual variability. Our results have shown for the first time that PRP-clots can be produced with higher or lower mechanical properties depending on the number of platelets in a patient blood. Patients with higher platelet number produce a clot with a higher density of fibrin fibers that give higher resistance to fracture in compression tests. Semiquantitative results of hOB adhesion to the PRP-clots and hOB migration inside them show that clots obtained from group A (poorer in platelets) are favored to host cells probably due to the lower fibrin density that allows cells to migrate and proliferate easier within the clot. It is known that there is a link between structure and cell movement beyond the effects of pore size of the scaffold so we think it will be interesting to study better cell behavior with different PRP clots. Geometrical and mechanical properties of scaffolds are able to influence the cell behavior and their response to differentiating stimulations [23,24]. The presence of aligned collagen structures has often been observed to improve cell motility, with suggested mechanisms including mechanical anisotropy, contact guidance, and molecular-scale topography [25,26]. Clots obtained from patients with higher platelet number were expected to have higher biologic activity due to higher growth factors although research showing this does not appear to have been reported in the literature. The clots we obtained from both groups of patients promote migration and proliferation of hOB in comparison with cells treated without PRP but without statistically significant differences between the two groups, indicating the same biological activity and potential for bone regeneration.

Aggregation and activation of platelets results in a rapid release of LPA into the extracellular environment [16,17] so we think that, when using PRP in bone regeneration, the potential role given by LPA should also be considered. With our results, we highlighted for the first time the presence of LPA in the PRP. In recent articles, we show, along with other researchers, that LPA is a promoter of osteoblasts proliferation [17] and that it stimulates osteogenesis of human mesenchymal stem cells, induces the expression of osteoblast marker genes [27], and increases the mineralization of osteoblasts [28]. LPA is believed to be a factor involved in the regulation of osteogenesis and in bone remodeling by inducing osteoblasts’ synthesis of osteoinductive cytokines and by committing MSCs towards osteoblasic differentiation [29,30]. Recent in vitro and in vivo studies have shown that LPA is present at elevated levels at sites of tissue injury and inflammation as a product of activated platelets [30] and is produced also by bone cells, thus identifying its potential for direct effect on various cellular activities that could involve control of bone mass and/or composition [13]. Together with its currently highly studied role in bone cell biology and bone regeneration [27], we wanted to emphasize that it could play a role as important as the growth factors already widely studied in the PRP. Applying PRPs in anatomical sites particularly at risk of infection such as maxillary sinuses and cutaneous wounds with frequent inflamed tissues or conditions of oxidative stress, we think that a possible role of LPA in such situations has to also be taken into account. About bacterial infection of the oral cavity, it is scientifically confirmed that surface geometry and microarchitecture of materials modified eukaryotic cell adhesion [31] and cariogenic streptococci adhesion [32]. Moreover, the corrugated plaques together with the altered texture of the mucosa create the right conditions for the colonization and the development of microbial species such as saprophytic bacteria or fungal species [33]. The development of novel biomaterial coatings or scaffolds that enhance early osseointegration yet deters the attachment of bacteria are especially desirable properties to avoid aseptic and septic loosening. About twenty years ago, Laux et al. [34] described how palmitoyl LPA inhibited the growth and virulence of *P. aeruginosa* and that exposure of bacteria to this lipid made them increasingly susceptible to certain antibiotics, suggesting LPA/LPA analogues as potential adjuncts in a bone regenerative setting to reduce potential infection risk of implantable devices. Only a few studies have been done on the activity of LPA on bacteria, and relatively scattered data suggest an important contribution of the platelet lipid signaling to sepsis [35] to consider its application as coating of dental or fixing bone implants [36]. LPA anti-infective role deserves to be more widely studied together with its effect in inflamed tissues of the oral cavity and systemic conditions related to oxidative stress impairing. We found that this lipid by-product of autotaxin activity is involved in cancer, vascular defects, and neural tissue but is largely unexplored in the immune system. In blood vessels, LPA enhanced neo-intimal hyperplasia following vascular injury by modulating proliferation, autophagy, inflammation, and oxidative stress and may contribute to the pathology of atherosclerosis [37] whereas, in liver, can block the pathogenesis of acute liver injury decreasing inflammatory cytokines [38]. In glial cells through its receptor, LPA protects from oxidative stress [39] and exerts antiaging effect in age-related diseases by improving the anti-oxidative ability of yeast cells [40]. Few results support the LPA role during infection or inflammation, and more studies deserve to be done considering its role in human macrophage formation with PPARγ as the key master regulator. As the macrophages are important producers of biologically active molecules, LPA-mediated macrophages could participate in both beneficial and detrimental outcomes in inflammation of the site where PRP, rich in LPA, will be used [41]. Macrophages have shown to be key players during tissue regeneration, wound healing, and prevention of infection, and they contain antimicrobial effects that are capable of reducing bacterial contamination after surgeries. Osteomyelitis, for example, is commonly reported in 9.5% of wisdom tooth removal, and when a PRP-Clot plug was inserted after extraction, this was significantly reduced to 1% of cases [42]. Despite these reported findings, very little is already known about what the antibacterial properties of PRP are, as very few studies have investigated this phenomenon.

Considering that no higher LPA concentrations were found in the clots of the two groups but only in the non-clotted fractions (in the residues), they probably are due to different biological effects. This result is not seen in Figure 5 because the experiment has been done using the clots. In conclusion, we think that liquid F2 fraction should to be used in vivo together with the clots, considering them only as a scaffold with a mechanical property. In addition, our results clearly showed that plasma from patients higher in platelets had higher LPA basal levels, so we asked ourselves if the number of platelets in patient’s blood could influence pharmacological effectiveness of PRP in tissue regeneration applications. Platelet-rich plasma (PRP) preparation is widely used and has rapidly grown up in orthopaedic practice. It is accepted that the bone healing process is mediated by numerous biomolecules and involves the hematoma formed at the fracture site. Moreover, our results showing that centrifugation, conventionally used to concentrate plasma to F1 and F2 fractions and then to activate it to produce a clot, did not alter LPA quantity, opened a discussion about the role of PRP as a pharmacologically active 3D scaffold. According to our quantitative data obtained on LPA, we can postulate that the level of growth factors can be influenced by the basal number of platelets in patient blood but not by the centrifugation done to obtain F1 and F2 or activation in a clot. The centrifugation may be used only to influence the clot’s mechanical properties by concentrating platelets, but this process seems not to increase the number of growth factor that will be released. This consideration should be taken into account, especially because, in bibliography, many of the studies done just quantify growth factors in PRP-F2 after centrifugation and concentration of platelets. Accordingly, they highlight a correlation between the higher platelet concentration obtained after centrifugation and growth factor released [43,44]. Results obtained in this way are widely accepted, but maybe, a further analysis should be required in order to obtain more reliable data [45]. Certainly, a comparison of the growth factors’ quantification between plasma, F1, F2, clot’s lysate, and residues, as we did, could reveal new aspects related to PRP treatment. Therefore, growth factor levels involved in bone regeneration need to be quantified both in blood and in all the different fractions used in clinic to better correlate their role in tissue regeneration and patient outcomes.

In addition, LPA release time from gels is an important factor that should be considered indeed; we doubt that bioactive phospholipid can be released by α-granules like other growth factors, and therefore, platelet degranulation could not be enough to release it. It may be necessary to have a biological production time longer than the sixty minutes for coagulation. This data is supported by the small difference occurring in LPA concentration among the plasma, the supernatants, and the non-coagulated portion.

The clinical implications of the present study’s results are potentially fundamental for the everyday bone regenerative medicine and may be useful for the clinical operator in order to create a scaffold with adjustable properties to each situation. In fact, the possibility that patients with a higher number of platelets could “produce” PRP-clots with a higher density of fibrin fibers and, consequently, with a higher resistance to fracture in compression tests is a crucial point. A better outcome in these patients could also be due to higher levels of growth factor in PRP liquid fraction that probably will be the real responsibility of the biologic activity. This could explain the various outcomes of bone regeneration in different patients. It could eventually determine a relative contraindication of the PRP technique in patients with low level of platelets, or it could become selection criteria for the use of PRP in regenerative medicine.

## 4. Materials and Methods

### 4.1. Sample Preparation

Venous blood from healthy male and female volunteers aged between 20 and 60 years was obtained and used for the preparation of the platelet-rich plasma (PRPs) samples. Inclusion criteria were ages between 20 and 60 years; nonsmokers; and absence of chronic hematologic, neoplastic, and/or infectious diseases (HIV+, HCV+, and HBV+). All procedures involving human participants were performed in accordance with the ethical standards of the institutional research committee and with the 1964 Helsinki Declaration and its later amendments or comparable ethical standards. All subjects gave their informed consent for inclusion before they participated in the study. The protocol was approved by the Ethics Committee of Novara “Prot. CE-Novara62/2018”, approved on 27 July 2018.

Samples of collected blood were analyzed using a hematology autoanalyzer Sysmex XN-2000™ (Sysmex, Kobe, Japan) to obtain a platelet count. Samples were then centrifuged at 2500 rpm for 8 min at 25 °C without acceleration and without break in order to obtain PRP in the upper half of the tube, separated from the remaining portion of red blood cells by a thin layer of buffy coat. The PRP portion was removed as two fractions of equal volume: the more superficial one, called fraction 1 (PRP-F1), and the part nearest to the leukocyte fraction, called fraction 2 (PRP-F2). Platelet counting of PRP-F1 (F1) and PRP-F2 (F2) was performed using the Sysmex XN-2000™ before placing F2 into sterile vials and activating it according to Endoret^®^ kit using 10% CaCl_2_ at 37 °C for 1 h to create the platelet concentrate (F2-clot). The obtained clots were analyzed using macroscopic, mechanical, and microscopic methods. Moreover, the in vitro activity of the PRP clots on proliferation and migration of osteoblast-like cells was assessed together with LPA quantification that was done on PRP-F1 (F1), PRP-F2 (F2), and Endoret^®^-activated PRP-F2 (F2-clots).

Plasma concentrates (F2-clots) prepared from PRP from patients with low platelet number (group A) and PRP from patients with high platelet number (group B) were compared.

### 4.2. Macroscopic and Mechanical Observations

Macroscopic images of the obtained F2-clots were obtained. The compression properties of clotted PRPs were tested using an Electro Force BioDynamic Test Instrument (Bose; TA Instruments, Eden Prairie, MN, USA). Samples of 2 ± 0.5 mm^3^ were positioned inside the instrument’s chamber between two pistons. Tests were performed with a constant crosshead speed of 0.05 mm/s. The instrument showed the real-time displacement of the piston and the force acting on the sample, producing a force/displacement curve. Compressive strength was then calculated, using the surface area of the sample, the initial length of the sample, compressed length, and force at breaking point. All samples were tested in triplicate, and data are presented as mean ± standard deviation (SD), comparing F2-clots from patients of group A and patients of group B

### 4.3. Microscopic Observation

SEM analysis of the coagulated platelet plasma concentrates was performed. After cacodylate buffer washing, F2-clots were fixed in a 4% paraformaldehyde +2.5% glutaraldehyde solution in 0.1 M cacodylate buffer at pH 7.4 for 30 min at 4 °C. Then, samples were dehydrated using a series of solutions of increasing ethanol concentration (50% to 100%) and, afterwards, in hexamethyldisilizane (20). Finally, they were glued onto aluminum stubs using a colloidal silver fluid, gold sputtered (Balzer, Bal-Tec SCD 004), and analyzed by electron microscopy (Quanta 200 FEI Philips SEM, ThermoFisher Sci., Hillsboro, OR, USA).

### 4.4. In Vitro Test

Bone trabecular fragments were taken during surgical interventions from patients in accordance with the ethical standards of the institutional research committee and with the 1964 Helsinki Declaration and its later amendments or comparable ethical standards. All subjects gave their informed consent for inclusion before they participated in the study. The protocol was approved by the Ethics Committee of Novara “Prot. CE-Novara 62/2018 on 27 July 2018. Fragments were digested using collagenase/elastase as described previously [17] to obtain hOB. Where not specified, reagents were from Sigma-Aldrich (Milan, Italy). The osteoblasts were cultured in Iscove’s modified Dulbecco’s medium (IMDM; EuroClone, Milan, Italy) supplemented with 10% fetal calf serum (Hyclone, from EuroClone, Milan, Italy), 2 mM l-glutamine, and antibiotics. Cells from up to five passages were used for all the experiments. Prior to culturing cells in the presence of a PRP test sample, cells were starved for 24 h and cultured in serum free media supplemented with 250 mg/mL essentially fatty acid-free human serum albumin.

Proliferation was measured using a luminescent-based ATP quantification Kit (ViaLight Cambrex Profarmaco, Milan, Italy). Cells were lysed using the cell lysis reagent and treated with ATP monitoring reagent at time 0 and following 1, 3, 6, and 12 days of culture of cells with PRP. The light produced was measured in a luminometer and expressed as relative luminescence units (RLUs).

To determine the migration activity of cells in response to PRPs, 1.5 × 10^5^ cells were plated in 24-well plates in 500 μL of medium and incubated at 37 °C in 5% CO_2_ until confluence; then, serum was starved for 20–24 h. Monolayers were wounded by the introduction of linear scratches with a sterile pipette tip, rinsed with PBS (phosphate saline buffer pH 7.4), and cultured in medium with and without the tested PRP groups. For each experiment, cells in six selected fields were tracked for their distance travelled. The selected fields (30–40 µm) were maintained at a fixed distance from the border of the culture plate, and for each experiment, images were acquired using a Leica digital camera connected to an inverted microscope (Leica). Images were obtained at time 0, 6 h later, and 1–3 days after the addition of the PRP. The distances travelled by the cells were measured using the Qwin Image Analysis system (Leica).

To observe the adhesion and migration of cells in PRPs, clots were placed in the wells of a low adhesion multi-well plate with 1 mL of IMDM containing 10^5^ hOB. The multi-well was then put in a shaking incubator (SanyoCO2) at 37 °C, 5% of CO_2_, and 80–85% humidity. Following 48 h of incubation with hOB, PRP clots were treated with DAPI (4′,6′-diamidino-2phenylindole), which marked the hOB DNA, so that cell penetration could be estimated using fluorescence microscopy.

### 4.5. LPA Quantification

A commercial enzyme-linked immunosorbent assay (ELISA) kit was used for LPA measurement (E3406Hu, Li StarFish, Milan, Italy) according to the manufacturer’s instructions. Briefly, standards and samples (50 μL) were aliquoted to the pre-coated plates in duplicates. After 1 h of incubation and washing, 50 μL of conjugate was added. After 1 h of incubation and washing, 100 μL of enzyme substrate was added to each well and incubated for 15 min before adding 100 μL of solution. The optical density of each microwell was read using a microplate reader at 450 nm, and the level of LPA in each well was calculated using a logarithmic standard curve (requiring an *R*^2^ value of above 95%); the average of the duplicates was used as the results.

Obtained data were reported in μg/mL.

### 4.6. Statistical Evaluation

An unpaired Student’s *t*-test was used for the statistical analyses of clot mechanical properties. Cell proliferation differentiation and motility results were analyzed using Bonferroni Student’s two tailed *t*-test (SPSS, Chicago, IL, USA) to compare the groups. Statistical significance was set at *p* < 0.05. All test samples were assayed in duplicate, and experiments were repeated between two and four times.

## 5. Conclusions

More studies are needed to better address the application of the clinical work and to deepen how the morphologic characteristics of clots can affect the patient’s outcome. According to the literature, clots’ morphological and structural differences may influence cell homing and clot interaction with the surrounding environment. Certainly, more detailed studies on PRP-clots as scaffolds will be required. Three-dimensional structural tests on fiber orientation, pore dimensions, and mechanical properties are only some variables that need more attention. Even if no clinical conclusions can ever be drawn from an in vitro study, a scaffold with low mechanical properties can be considered a good bone substitute only for clinical situations that did not require esthetic and functional outcomes and that require low time of permanence such as the filling of wide pneumatized sinus lift [46]. An in vivo model could be used to understand better how the different PRP-clots obtained from patients rich or poor in platelets and added with PRP soluble form interacts and integrates with the surrounding area, creating a network through the growth factors released.

The role of LPA should also be evaluated further in future research, as it constitutes a promising PRP research topic. Moreover, as the presence of LPA in PRP had never been highlighted before, in the same way, further research could lead to the discovery of other new growth factors correlated to PRP.

Our results, in spite of the evident need for further confirmations and research, represent an important step for an improved understanding of PRP use for bone regeneration and for its more appropriate clinical use.

## Figures and Tables

**Table 1:** Table reporting mean ± SD of the platelet number in the two groups (group A, patients with lower platelet number; group B patients with higher platelet number) and in the PRP fractions F1 and F2 obtained following centrifugation.

CELLS/μL	GROUP A	GROUP B
PLASMA	114,500 ± 35,500	232,600 ± 35,500
F1	82,100 ± 2500	135,200 ± 38,200
F2	152,000 ± 36,000	313,200 ± 79,400

**Table 2:** Table reporting the mean ± SD; *n* = 16 of lysophosphatidic acid (LPA) quantification in the two groups (group A, patients with lower platelet number, and group B, patients with higher platelet number). The table also reports LPA levels in the two plasma PRP fractions F1 and F2 obtained following centrifugation. F2-clot represents LPA levels in lysates of clots obtained from F2 phase, while the F2 clot-residual represents LPA quantification in F2 liquid residual after clotting with CaCl_2_. * *p* < 0.05 between the two groups.

LPA μg/mL	PLASMA	F1	F2	F2-Clot	F2 Clot-Residual
GROUP A	5.17 ± 0.05	5.23 ± 0.08	5.19 ± 0.03	2.72 ± 1.01	4.98 ± 0.15
GROUP B	7.42 ± 0.04 *	7.39 ± 0.05 *	7.46 ± 0.03 *	2.64 ± 1.20	7.14 ± 0.24 *
